# Key Determinants of Cardiovascular Outcomes in Multi‐Ethnic Patients With Rheumatic Disease Using JAK Inhibitors

**DOI:** 10.1002/msc.70066

**Published:** 2025-02-15

**Authors:** Kehinde Sunmboye, Ahsan Memon, Maumer Durrani

**Affiliations:** ^1^ Rheumatology Department University Hospitals of Leicester Leicester UK; ^2^ University of Leicester Leicester UK

**Keywords:** cardiovascular events, cardiovascular risk, JAK inhibitors, multi‐ethnic cohort, rheumatic diseases, social deprivation

## Abstract

**Background:**

Janus kinase (JAK) inhibitors are effective therapies for autoimmune rheumatic diseases (ARDs), but concerns persist regarding their cardiovascular effects, particularly in diverse patient populations. Identifying determinants of cardiovascular risk is essential for optimising therapy and outcomes, especially in multi‐ethnic cohorts.

**Objective:**

To assess clinical and socioeconomic determinants, including age, deprivation decile and ethnicity, in predicting cardiovascular events among patients on JAK inhibitors in a multi‐ethnic cohort.

**Methods:**

A retrospective cohort study of 309 patients with ARDs (mean age 59.3 years, 77% female, 73% White, 25% South Asian) receiving JAK inhibitors at a UK teaching hospital was conducted. Cardiovascular events, including myocardial infarctions, strokes and cardiovascular‐related deaths, were recorded. Multivariate logistic regression assessed associations between age, deprivation decile, ethnicity and cardiovascular outcomes.

**Results:**

The combined effect of age and deprivation decile significantly predicted cardiovascular events (*p* = 0.031). Older age demonstrated an odds ratio (OR) of 1.06 (95% CI: 1.00–1.13). Neither age nor deprivation decile alone achieved statistical significance, but their combination provided a robust model with an AUC of 0.837. Ethnicity was not independently predictive in this cohort.

**Conclusions:**

In a multi‐ethnic cohort, age and deprivation decile jointly predict cardiovascular events in patients on JAK inhibitors. Socioeconomic factors should be integrated into cardiovascular risk assessment models to inform personalised care strategies for patients receiving JAK inhibitor therapy.

## 1 Introduction

Cardiovascular disease (CVD) remains a leading contributor to morbidity and mortality globally and poses significant challenges for both prevention and management (Collins and Altman 2010). Patients with autoimmune rheumatic diseases (ARDs), including rheumatoid arthritis (RA), psoriatic arthritis (PsA) and ankylosing spondylitis (AS), experience a notably higher cardiovascular risk compared with the general population (Alhusain and Bruce 2013). This increased burden is multifactorial, arising from systemic inflammation, shared traditional risk factors such as hypertension and dyslipidemia, and the effects of immunomodulatory therapies (Yates et al. 2021).

Janus kinase (JAK) inhibitors have emerged as targeted synthetic therapies for ARDs, offering significant improvements in disease control and quality of life (Solipuram et al. 2021). However, their use has raised concerns regarding potential cardiovascular adverse effects, particularly in high‐risk populations with underlying comorbidities (Wei et al. 2023; Kimenai et al. 2022). Evidence on this issue remains inconclusive, with some studies suggesting increased cardiovascular risks, while others highlight a need for more robust, large‐scale analyses (Kimenai et al. 2022; Lang et al. 2016).

Beyond clinical factors, non‐clinical determinants such as socioeconomic status have also been recognized as critical predictors of cardiovascular outcomes. Deprivation indices, often derived from socioeconomic variables, serve as indicators of healthcare disparities, lifestyle factors, and access to care (Lang et al. 2016; Cavallari et al. 2018). Individuals from more deprived socioeconomic backgrounds frequently exhibit higher rates of cardiovascular disease, attributed to cumulative exposure to risk factors, delayed healthcare access, and poorer preventive care (Hippisley‐Cox, Coupland, and Brindle 2014).

Age is another well‐established independent predictor of cardiovascular events. The incidence of CVD increases significantly with advancing age due to the progression of atherosclerosis, vascular stiffness and other age‐related changes (Rodgers et al. 2019). In patients on biological therapies, age has been linked to varying cardiovascular outcomes, yet its role alongside JAK inhibitors remains an area of ongoing investigation (Borren and Ananthakrishnan 2019). Interestingly, some studies suggest that JAK inhibitors may not increase cardiovascular risk compared to other therapies (Solipurum et al, 2021; Kotyla, Islam and Engelmann 2020).

Given these uncertainties, further research is necessary to clarify the determinants of cardiovascular events in patients receiving JAK inhibitors, particularly within diverse and multi‐ethnic populations. Ethnicity itself may influence cardiovascular risk, as shown in prior studies where distinct ethnic groups exhibited differential CVD outcomes and risk prediction model performance (Mu et al. 2022; Tillin et al. 2014).

This study aimed to evaluate the combined impact of clinical and socioeconomic factors, including age, deprivation indices and ethnicity, in predicting cardiovascular outcomes among a multi‐ethnic cohort of patients receiving JAK inhibitors for ARDs. By addressing these determinants, we hope to provide insights that enhance cardiovascular risk assessment and inform personalised care strategies.

## 2 Materials and Methods

### 2.1 Study Design and Setting

This study employed a cross‐sectional retrospective cohort design to evaluate cardiovascular outcomes in patients diagnosed with rheumatoid arthritis (RA), psoriatic arthritis (PsA) or ankylosing spondylitis (AS) who were receiving JAK inhibitor therapy. Data were collected from three hospital sites under a single NHS University Teaching Hospital in the United Kingdom. The study period spanned from May to July 2023.

### 2.2 Participants, Data Sources and Collection

Patient data were retrieved from a biologics database, a comprehensive repository of individuals with rheumatic diseases undergoing biological or targeted synthetic therapies. Extracted baseline characteristics included demographic variables (age, gender, ethnicity), diagnosis, family history of cardiovascular disease, lipid profiles, comorbid conditions and calculated Q‐risk (QRISK3) scores. Socioeconomic status was measured using deprivation deciles.

Patients eligible for inclusion were identified using a standardized search algorithm that filtered for individuals with ≥ 12 months of follow‐up data while on JAK inhibitor therapy. Cardiovascular events—defined as angina, myocardial infarction, stroke, or cardiovascular‐related deaths—were retrospectively identified through standardized diagnostic coding systems.

All patients provided prior consent for inclusion in the biologics database and data were utilised in adherence to institutional research ethics protocols.

### 2.3 Eligibility Criteria

- **Inclusion Criteria:** Adults (≥ 18 years) with a confirmed diagnosis of RA, PsA, or AS who were actively receiving JAK inhibitor therapy during the study period.
- **Exclusion Criteria:** Patients with pre‐existing cardiovascular disease were excluded although no such cases were encountered in this cohort.

#### 2.3.1 Outcomes and Exposures

The primary outcome was the occurrence of cardiovascular events, dichotomised as either present or absent. Events included angina, myocardial infarction, stroke and cardiovascular‐related mortality.

#### 2.3.2 Independent Variables

Key independent variables included

- Age
- Gender
- Ethnicity
- Diagnosis (RA, PsA, AS)
- Family history of cardiovascular disease
- Lipid profiles
- Comorbidities (hypertension, diabetes, dyslipidemia)
- Socioeconomic status (deprivation decile)
- Q‐risk (QRISK3) score

No significant effect modifiers were identified.

#### 2.3.3 Outcome Definition and Cardiovascular Event Identification

Cardiovascular events were coded according to ICD‐10 standards:

- **Angina:** I20
- **Myocardial Infarction:** I21
- **Stroke:** I63
- **Cardiovascular‐related deaths:** I11, I13, I20‐I25, I27‐I28 and I50.

#### 2.3.4 Statistical Analysis

Statistical analyses were conducted using the DATAtab online statistical platform. Multivariate logistic regression was applied to evaluate associations between predictor variables and cardiovascular events. The regression model was adjusted for potential confounders, including gender, ethnicity and comorbidities.

Independent variables were analysed both individually and in combination to assess their predictive value. Statistical significance was determined using a *p*‐value threshold of < 0.05.

#### 2.3.5 Model Development and Evaluation

A multivariate logistic regression model was built using a backward selection method, where all covariates were initially included and non‐significant variables were iteratively removed. Model fit was assessed using the Pearson chi‐squared test, while discrimination was evaluated through the area under the receiver operating characteristic (ROC) curve (AUC).

#### 2.3.6 Quantitative Variables and Covariate Selection

Continuous variables, such as age and Q‐risk scores, were directly included in the regression analysis. Covariates were selected based on their potential to confound the relationships between predictors and cardiovascular events.

#### 2.3.7 Bias Minimisation

To mitigate bias, several strategies were implemented:

1. Use of a retrospective cohort design to minimise selection bias.
2. Inclusion of all eligible patients regardless of cardiovascular outcomes.
3. Adjustment for multiple confounding variables in the regression analysis.
4. Inclusion of a large, ethnically diverse population to enhance generalisability.

### 2.4 Additional Methods

- **Q‐risk Calculation:** Cardiovascular risk scores (QRISK3) were calculated using the online tool at qrisk.org.
- **Deprivation Decile:** Socioeconomic deprivation scores were derived using the English Index of Multiple Deprivation (IMD) postcode checker at fscbiodiversity.uk/imd.

#### 2.4.1 Sensitivity Analysis

To validate findings, a sensitivity analysis was performed by excluding patients with a prior history of cardiovascular disease. The association between individual and combined predictor variables with cardiovascular events was then reassessed.

## 3 Results

### 3.1 Descriptive Statistics

A total of 309 patients with a mean age of 59 years (SD +/− 12.3 years). The majority were female (77%). Table 1 shows that 73% of the study cohort were White, with 25% of South Asian ethnic origin. Only 0.6% were of Black ethnic origin. Table 1 also provides the demographic characteristics of the mean age of the study participants.

**TABLE 1:** Patient mean age and ethnicity with minimum and maximum age.

Ethnicity	Frequency	Percentage %	Mean age (years)	Standard deviation	Minimum age (years)	Maximum age (years)
White	228	73%	60.04	12.35	19	87
South asian	79	26%	57.61	12.41	20	87
Black	2	1%	55.5	0.71	55	56

**FIGURE 1:**
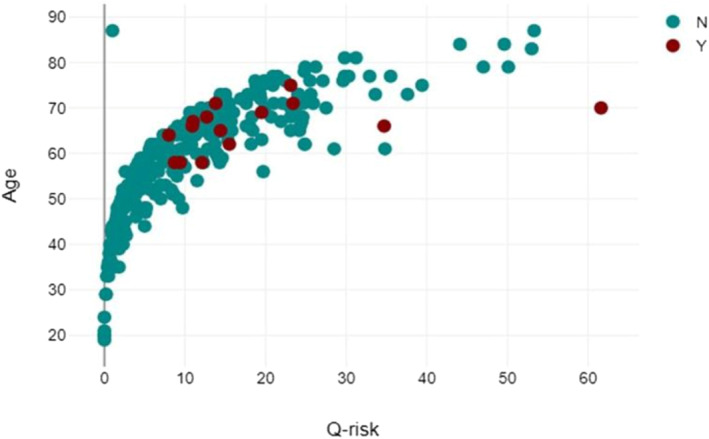
Scatter diagram showing the Q‐risk (QRISK3) value and cardiovascular events by age.

Figure 1 shows via scatter diagram, the distribution of the Q‐risk by age. The median Q‐risk (QRISK3) score was 11.54% (range: 0%–62%), indicating a moderate cardiovascular risk.

Out of the 309 patients observed, there were only 14 events. There were no missing data, so all 309 patients were used in the final analysis. Table S1 (see supplementary material) shows a 2 × 2 table showing the records of cardiovascular events in patients on the biologics database on JAK inhibitor therapy.

**FIGURE 2:**
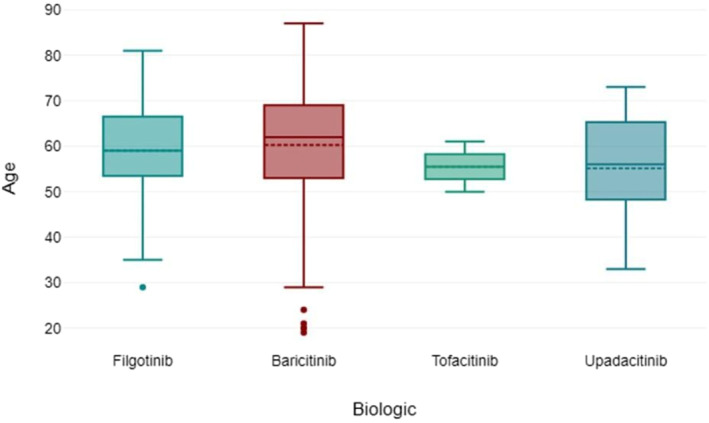
Age of patient by JAK inhibitor therapy used.

**FIGURE 3:**
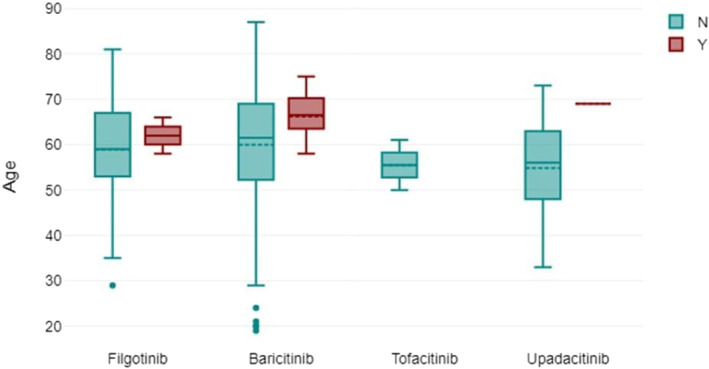
Box plot showing the incidence of cardiovascular events by age and JAK inhibitor therapy.

**FIGURE 4:**
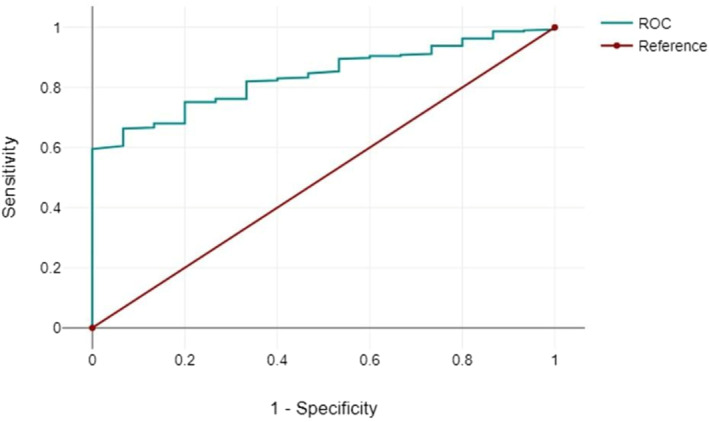
ROC Curve of the logistic regression model. Area under the curve (AUC) of 0.837.

Figure 2 shows the age distribution of the patients via box plot by the type of JAK inhibitor used. Figure 3 shows via box plot that there was no numerical or statistical significance in the number of cardiovascular events resulting from a type of JAK inhibitor therapy. Figure 4 shows JAK inhibitor use by disease subtype distribution.

The study population was predominantly from a deprived area with a deprivation decile of 3 (1 = most deprived; 10 = least deprived), with the majority (13%) of participants belonging to this deprived deprivation decile. Around 7% of the participants were from the most deprived areas (deprivation decile 1) and 10.2% were from the least deprived areas (deprivation decile 10).

### 3.2 Inferential Statistics: Use of the Age of the Patient and Deprivation Decile as Independent Variables

Multivariate logistic regression analysis was performed to determine the independent predictors (age and deprivation decile) for cardiovascular events while adjusting for potential confounders including gender, ethnicity, disease duration and co‐morbidities. Tables S2 and S3 show that the regression model and model summary were statistically significant (see supplementary material).

The age of the patient and deprivation decile (independent variables) in the regression model highlighted the risk of cardiovascular events in patients on the various JAK inhibitor therapies. The results revealed in Table 2 show a statistically significant model using both the age of the patient and the deprivation decile together.

**TABLE 2:** *p*‐value, odds ratio, and 95% confidence interval of the independent variables using logistic regression analysis.

	Coefficient *B*	Standard error	*Z*‐value	*p*‐value	Odds ratio	95% confidence interval
Constant	−6.89	2.24	3.08	0.002	0	0–0.08
Baricitinib	0.35	0.83	0.42	0.675	1.41	0.28–7.17
Tofacitinib	−18.93	51994.71	0	1.00	0	Unbounded
Upadacitinib	0.02	1.3	0.02	0.987	1.02	0.08–12.95
Filgotinib	0	1.00	0	0.896	1.00	Unbounded
Deprivation decile	0.43	0.97	0.45	0.654	1.54	0.23–10.25
Age	0	0	1.00	0.499	1.00	Unbounded
Age and deprivation decile	0.06	0.03	2.05	0.04	1.06	1–1.13

Figure 4 shows the ROC curve of the logistic regression model.

A positive correlation was observed between higher Q‐risk (QRISK3) scores and greater deprivation levels, highlighting an increased cardiovascular risk among patients from more socioeconomically deprived areas. The multivariate logistic regression model demonstrated statistical significance (Chi^2^(14) = 24.04, *p* = 0.031, *n* = 309). When age and deprivation decile were analysed together, they emerged as significant predictors of cardiovascular events in patients on JAK inhibitor therapy (OR: 1.06, 95% CI: 1.00–1.13, *p* = 0.04).

Individually, neither age nor deprivation decile showed statistical significance; however, their combined effect strengthened the model. The coefficient for age (*b* = 0.06) was positive, and its corresponding *p*‐value (0.04) confirmed statistical significance. The model demonstrated good discriminative ability with an area under the curve (AUC) of 0.837.

Further analysis assessed the impact of specific JAK inhibitors—baricitinib, filgotinib, tofacitinib and upadacitinib—on cardiovascular risk. None of the individual therapies showed a statistically significant association with cardiovascular events in this cohort. Similarly, the coefficients for deprivation deciles (ranging from 1 to 10) remained non‐significant.

## 4 Discussion

This retrospective study investigated the combined impact of age and deprivation decile on cardiovascular outcomes in patients receiving JAK inhibitor therapy for autoimmune rheumatic diseases. A significant association was identified when age and deprivation decile were analysed together (*p* = 0.04), underscoring the importance of incorporating socioeconomic factors alongside clinical variables for a more comprehensive assessment of cardiovascular risk. Interestingly, neither age nor deprivation decile individually demonstrated statistical significance, reinforcing the need to evaluate these factors in combination.

Our findings have notable implications for clinical practice. First, they highlight that age alone may not be a sufficient predictor of cardiovascular risk in patients treated with JAK inhibitors. Second, socioeconomic factors, such as the deprivation decile, play a critical role in cardiovascular outcomes. Patients from deprived backgrounds often encounter challenges such as reduced healthcare access, higher comorbidity burden, and poorer cardiovascular risk management (Kotyla, Islam, and Engelmann 2020). Incorporating these factors into predictive models allows for a more tailored and equitable approach to risk stratification.

The lack of significant findings when analysing Q‐risk (QRISK3) scores in isolation is particularly noteworthy. While QRISK3 remains a validated tool for cardiovascular risk estimation, it may not fully account for the unique complexities observed in patients with autoimmune conditions receiving JAK inhibitors (Mu et al. 2022). This suggests that additional variables, such as socioeconomic deprivation, should be considered to enhance the accuracy of risk prediction models. Healthcare providers should therefore integrate both clinical and socioeconomic determinants to better identify high‐risk patients and implement targeted preventive strategies (Tillin et al. 2014).

Our study also evaluated the role of specific JAK inhibitors, including baricitinib, filgotinib, tofacitinib, and upadacitinib, in influencing cardiovascular risk. None of the therapies demonstrated a significant association with cardiovascular events in this cohort. These results align with prior research suggesting that JAK inhibitors, as a class, do not inherently elevate cardiovascular risk compared with other therapeutic options (Sunmboye, Petrie, and Durrani 2023; Kotyla, Islam, and Engelmann 2020; Livingstone et al. 2021; Schofield, Crichton, and Chen 2012). This reinforces the importance of individualised treatment planning, ensuring that patients at elevated cardiovascular risk are closely monitored without unnecessary discontinuation of effective therapies (Sunmboye, Petrie, and Durrani 2023).

## 5 Strengths and Limitations

The study’s strengths include its relatively large sample size (*n* = 309) and the inclusion of a diverse, multi‐ethnic cohort (Caucasian, South Asian and Black participants), reflecting broader demographic patterns in the United Kingdom. The data were drawn from a comprehensive biologics database with a substantial patient catchment area exceeding one million individuals, enhancing the robustness of our findings.

However, certain limitations should be acknowledged. The retrospective design may introduce bias inherent to observational studies. Additionally, all three hospital sites were part of a single NHS University Hospitals Trust within the UK, which may limit the generalisability of our results to populations outside this region or internationally.

## 6 Interpretation

Our findings demonstrate that age and deprivation decile, when assessed together, are independent determinants of cardiovascular events in patients receiving JAK inhibitors. Clinicians should incorporate these factors into cardiovascular risk assessments and closely monitor high‐risk patients to optimise outcomes.

## 7 Generalisability

Although our results are relevant to patients with rheumatoid arthritis, psoriatic arthritis and ankylosing spondylitis on JAK inhibitors in the UK, additional studies are needed to confirm these findings across different healthcare systems and populations. Future research should aim to validate these results in larger, multi‐country cohorts to ensure broader applicability.

## 8 Conclusions

This study underscores the critical role of both clinical and non‐clinical factors, particularly age and socioeconomic deprivation, in predicting cardiovascular events among patients with autoimmune rheumatic diseases treated with JAK inhibitors. By integrating socioeconomic determinants, such as deprivation decile, into cardiovascular risk assessment models, healthcare providers can improve risk prediction accuracy and tailor preventive strategies to address disparities in care. These findings advance the principles of personalised medicine and highlight opportunities for optimising cardiovascular risk management in this patient population.

To refine these insights further, future research should focus on identifying precise thresholds for age and deprivation deciles that maximise predictive utility. Developing more robust and validated risk models will facilitate the implementation of targeted, individualised preventive interventions. Additionally, prospective studies with larger, multi‐ethnic and geographically diverse cohorts are essential to confirm these results and strengthen their applicability across different clinical settings.

In conclusion, this study highlights the importance of incorporating socioeconomic factors alongside traditional clinical variables to enhance cardiovascular risk assessment. Doing so has the potential to improve health outcomes and reduce disparities for patients with autoimmune rheumatic diseases receiving JAK inhibitor therapies.

## Author Contributions

All authors reviewed the final version to be published and agreed to be accountable for all aspects of the work. Concept and design: K.S., A.M., M.D. Acquisition, analysis or interpretation of data: K.S., M.D. Drafting of the manuscript: K.S., A.M., M.D. Critical review of the manuscript for important intellectual content: K.S., A.M., M.D. Supervision: K.S.

## Ethics Statement

This study adhered to ethical principles by maintaining data confidentiality and participant anonymity, as approved by the Research Ethics Committee. Approved May 2023, Project ID: 13188.

## Consent

Human subjects: Consent was obtained or waived by all participants in this study. Animal subjects: All authors have confirmed that this study did not involve animal subjects or tissue.

## Conflicts of Interest

The authors declare no conflicts of interest.

## Supporting information

Supporting Information S1

## Data Availability

Anonymized data are available upon reasonable request in accordance with ethical guidelines.
