# Supplementary material for: Key Determinants of Cardiovascular Outcomes in Multi‐Ethnic Patients With Rheumatic Disease Using JAK Inhibitors
Source: Musculoskeletal Care. 2025 Feb 15;23(1):e70066. doi: 10.1002/msc.70066 (PMC11829614; doi:10.1002/msc.70066)
Supplement: Supplementary file 1 — Supporting Information S1 [file MSC-23-e70066-s001.docx]

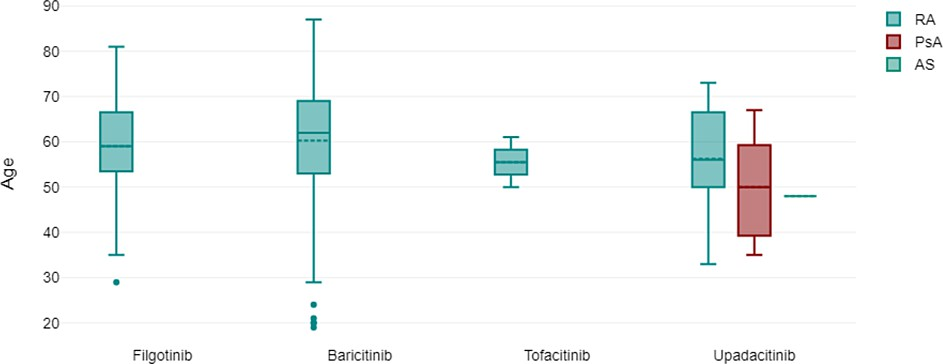


**Figure S1: Type of JAK inhibitor by rheumatic disease sub-type**

Abbreviations: RA=rheumatoid arthritis; PsA=psoriatic arthritis; AS=ankylosing spondylitis.

|  | **Yes** | **No** |
| --- | --- | --- |
| Predicted events | 0 | 309 |
| Observed events 14 295 | | |

**TABLE S1: 2 X 2 table showing the records of cardiovascular events in patients on biologics database on JAK inhibitor therapy.**

| **Chi2** | **Degrees of freedom** | **p-value** |
| --- | --- | --- |
| 24.04 | 13 | 0.031 |

**Table S2: Chi-square value for the logistic regression model.**

| **-2 Log-Likelihood** | **Cox & Snell R2** | **Nagelkerke R2** | **McFadden’s R2** |
| --- | --- | --- | --- |
| 95.98 | 0.07 | 0.23 | 0.2 |

**Table S3: Model summary values for the logistic regression model.**
